# Therapeutic observation of transcutaneous auricular vagus nerve stimulation for chronic migraine in pediatric patients: a case report

**DOI:** 10.3389/fpain.2025.1686043

**Published:** 2025-11-10

**Authors:** Siqi Weng, Yao Xue, Xuezhen Xiao, Berthold Hocher, Yishui Zhang, Xiaowei Yang, Qirui Liu, Yabin Ji

**Affiliations:** 1 Department of Neurology, Nanfang Hospital, Southern Medical University, Guangzhou, China; 2 Fifth Department of Medicine (Nephrology/Endocrinology/Rheumatology/Pneumology), University Medical Centre Mannheim, University of Heidelberg, Heidelberg, Germany; 3 Zhuhai Fudan Innovation Institute, Zhuhai, China; 4 Reproductive and Genetic Hospital of CITIC-Xiangya, Changsha, China; 5 IMD Institut für Medizinische Diagnostik Berlin-Potsdam GbR, Berlin, Germany; 6 Institute of Reproductive and Stem Cell Engineering, NHC Key Laboratory of Human Stem Cell and Reproductive Engineering, School of Basic Medical Science, Central South University, Changsha, China

**Keywords:** transcutaneous auricular vagus nerve stimulation, chronic migraine, pediatric patients, headache, non-pharmacological intervention

## Abstract

**Background:**

Although interest in migraine has increased in recent years, important gaps remain in understanding and optimizing its management. These gaps are particularly pronounced in pediatric migraine, which continues to be understudied.

**Case report:**

This case report demonstrates the efficacy and safety of transcutaneous auricular vagus nerve stimulation (taVNS) in an 8-year-old male patient with refractory chronic migraine with aura [two to three weekly episodes; visual analog scale (VAS): 5–9; duration of each episode was 24 h]. After discontinuing all prophylactic and abortive medications (except ibuprofen suspensions such as Motrin®), the patient underwent a 28-week taVNS protocol that involved the following phases: a 4-week acute intervention, a 4-week intermission period, a 12-week preventive intervention, and an 8-week follow-up. During the acute intervention phase, the patient’s headache duration decreased by 84.4%, and frequency was reduced to fewer than two episodes/week, with complete aura resolution. The preventive intervention yielded further improvement to fewer than 1 episode/week by week 8 (with a 37.5% reduction in medication use). At final follow-up, the patient maintained a medication-free status with only three mild episodes (VAS: 1–3; duration <30 min) in the last 4 weeks. No adverse events were observed.

**Conclusion:**

taVNS was effective and safe in the management of chronic migraine in the reported pediatric patient. These findings suggest the need for further evaluation of this non-pharmacological intervention in pediatric migraine.

## 1 Introduction

Headache disorders are the second leading cause of global disability (1, 2), with migraine being particularly prevalent. Chronic migraine substantially impairs daily functioning and quality of life (3, 4). Although most migraine research targets adults, pediatric migraine represents a significant health burden, causing notable disability and reduced quality of life. A meta-analysis of 48 pediatric studies estimated a pooled migraine prevalence of 11% (5). In China, 30.3% of high school students reported weekly headaches (6), while two studies found that 75.7% of 3,384 adolescents aged 10–18 experienced headaches annually (7, 8).

Therapeutic options for pediatric headaches remain limited. Conventional pharmacological treatments, such as triptans, ergotamines, and calcitonin gene-related peptide receptor antagonists, have raised safety concerns regarding developmental effects. Neuro-blockade offers an alternative but lacks sufficient evidence in adolescents (9–11). This gap highlights the need for safe, non-invasive, non-pharmacological therapies for pediatric migraine. Transcutaneous auricular vagus nerve stimulation (taVNS) electrically stimulates the auricular branch of the vagus nerve, modulating the autonomic nervous system and central pain pathways (12). Adult studies have demonstrated the efficacy and safety of taVNS in migraine prevention and treatment (13–17). Therefore, we hypothesized that taVNS may be a safe and effective treatment for pediatric migraine.

## 2 Case report

### 2.1 Baseline information

This study included an 8-year-old male patient who presented to our headache clinic with a 4-year history of recurrent headaches since May 2020. The headaches were characterized by dull and throbbing left temporal pain, accompanied by phonophobia, photophobia, nausea, and vomiting. Initially, attacks occurred over five episodes/year, lasted approximately 1 h, and resolved with sleep.

In November 2023, the patient was first diagnosed with migraine by a pediatric neurologist at the Guangzhou Women and Children's Medical Center. He was started on 5-hydroxytryptophan granules (5 g bid) and ergotamine tartrate tablets (15 mg qd). Investigations revealed a patent foramen ovale on echocardiography; EEG, head MRI, and blood biochemical tests were normal.

Despite treatment, the headaches persisted. In December 2023, flunarizine (2.5 mg qd) was initiated with partial improvement. However, by May 2024, the patient’s headaches worsened to 15–18 headache days per month, including 8–12 days with moderate-to-severe migraine features and additional days with milder migraine-type headache [visual analog scale (VAS): 1–3], lasting 24 h, accompanied by phonophobia, photophobia, nausea, vomiting, abdominal distension, and sleep disorders. Meeting the third edition of the International Classification of Headache Disorders criteria, the patient was diagnosed with chronic migraine in Nanfang Hospital, Southern Medical University. The pain intensity averaged VAS 5, rising to 8–9 at peak. An 8-mL dose of an ibuprofen suspension afforded only brief symptomatic relief.

The patient continued flunarizine (2.5 mg qd), 5-hydroxytryptophan (5 g bid), and ergotamine tartrate (15 mg qd), but found treatment unsatisfactory. He ultimately switched to an ibuprofen suspension (8 mL) PRN.

### 2.2 Enrollment intervention phase

#### 2.2.1 Acute intervention period

**Figure 1:**
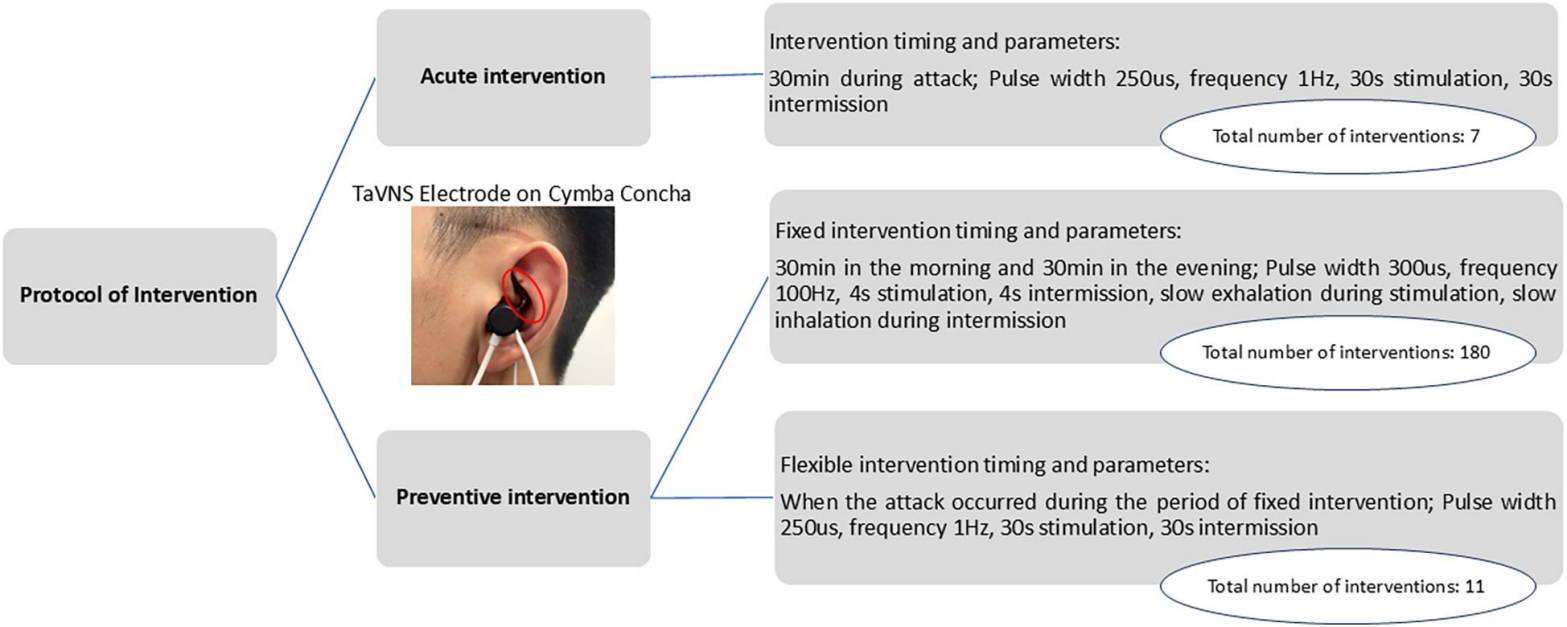
Intervention protocol, actual frequency of the intervention, and schematic diagram of a patient wearing the stimulation electrode (18).

The patient was enrolled in the study on 19 July 2024. The parameters for taVNS (BC102-IV, BrainClos Co., Ltd., Shenzhen, China) were a pulse width of 250 μs, frequency of 1 Hz (13), stimulation duration of 30 s, and interval of 30 s. The intensity was adjusted to the patient’s tolerance level without pain. The patient was instructed to use taVNS at home for 30 min during headache episodes (acute intervention) and to maintain a headache diary (Figure 1). A follow-up appointment was scheduled for 1 month later.

In the headache diary, the patient was required to record the VAS at the following six time points: during the attack, after treatment, 2 h after treatment, 8–12 h after treatment, and 24 h after treatment. VAS is a widely used method for assessing the effectiveness of migraine treatments.

At the follow-up on 22 August 2024, the headache diary data showed significant acute headache improvement (details in section 3.1). The family requested a 1-month stimulator discontinuation to assess symptom self-management. Upon follow-up on 21 September 2024, the patient reported a headache frequency of two episodes/week (duration 300–360 min; medication use once/week). The patient and family then reapplied for taVNS therapy and joined the taVNS Preventive Intervention project (18).

#### 2.2.2 Preventive intervention period

The patient was diagnosed with refractory chronic migraine. The preventive intervention project included the following two approaches: (1) TaVNS twice daily (morning/evening) for 30 min, with the following parameters: 300 µs pulse width, 100 Hz frequency (19, 20), and 4 s on/4 s off cycle. Additionally, the patient was instructed to exhale during stimulation and inhale during no stimulation. This synchronization enhances taVNS efficacy for pain intervention by more strongly activating the brain's pain inhibitory network (19, 20). (2) The second approach was flexible taVNS. The patients could use taVNS for an additional 30 min during headaches as an acute intervention, using the same parameters as those that were used in the acute intervention period (Figure 1). The 3-month treatment included monthly follow-ups, with the final assessment on 20 December 2024.

## 3 Results

### 3.1 Acute intervention

**Figure 2:**
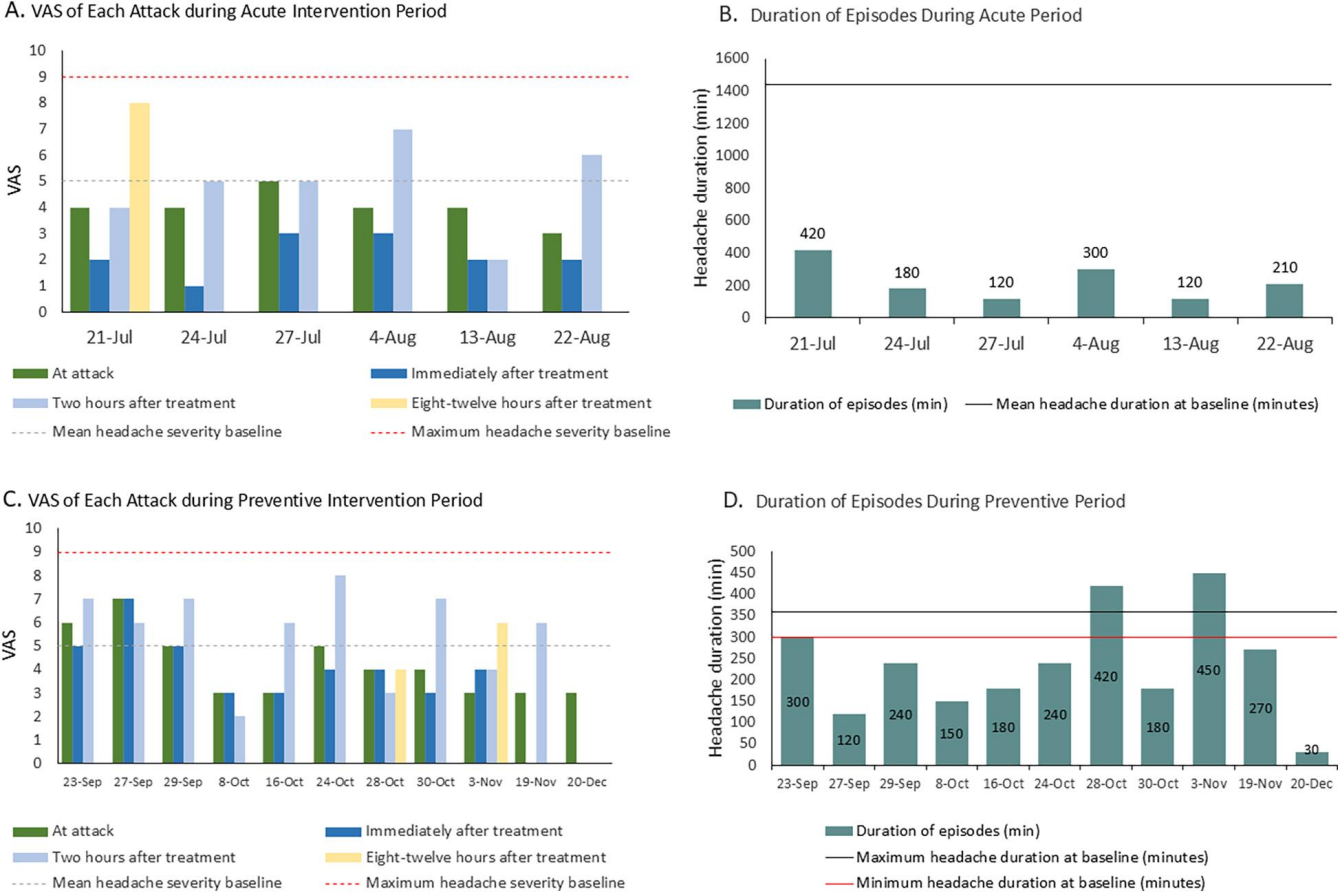
**(A)** Changes in VAS score during the acute intervention period. The VAS value for each bar represents the pain intensity at the corresponding time point. **(B)** Changes in the duration of headache episodes during the acute intervention period. **(C)** Changes in VAS score during the preventive intervention period. The VAS value for each bar represents the pain intensity at the corresponding time point. **(D)** Changes in the duration of headache episodes during the preventive intervention period.

The patient reported seven headache episodes 1 month after initiating taVNS (averaging <2 per week), with a mean duration of 225 min and a mean VAS pain intensity of 4.42. Headache intensity decreased by 1–3 points within 30 min following taVNS in six analyzable episodes (data were unavailable for one episode). Although headache recurrence occurred within 2–12 h, five of the six episodes resolved completely within 8–12 h. All the episodes had fully resolved by 24 h. An overall reduction in headache burden was observed over the 1-month period (Figures 2A,B), accompanied by a decreased use of medication (only four of seven episodes required medication). The patient’s associated symptoms also improved, as only one episode involved nausea/vomiting and no photophobia or phonophobia was reported. No taVNS-related adverse effects were observed/reported.

### 3.2 Preventive intervention

In the 3-month preventive intervention period, the patient reported 11 headache episodes. All were treated with flexible taVNS, with no headaches occurring during the fixed intervention sessions. Headache duration decreased significantly to a mean of 234 min (vs. 300–360 min pre-intervention), culminating in a final 30 min episode (Figures 2C,D). Attack intensity averaged VAS 4.2, with the last three episodes having an intensity of VAS 3. Frequency declined to <1 episode/week. Headache intensity decreased within 30 min post-taVNS in 50% of the episodes. The 2-h relief rate (4/11), namely, a reduction from a severe or moderate headache pain to a mild headache or none without the use of any rescue medication within 2 h after administering the investigated treatment, exceeded the acute intervention outcomes (1/6), demonstrating a downward severity trend. Medication was required for eight episodes. Motrin® was successfully tapered to 5 mL by 19 November. No nausea, vomiting, phonophobia, or abdominal distension occurred during this period. The patient reported a marked improvement in sleep disturbances.

### 3.3 Follow-up period

Following taVNS discontinuation (December 22, 2024), the patient's family was instructed to monitor his symptoms until 12 February 2025, with a scheduled 8-week follow-up on that day. Headache frequency decreased from 1 to 2 episodes/week (Follow-up W1–W2) to 3 mild episodes total (Follow-up W5–W8). Duration reduced from 120–180 min/episode to <30 min/episode. VAS scores declined from 4–5 to 1–3. Medication use transitioned from Motrin® 5 mL (2 total doses) to complete cessation. No associated symptoms recurred during the observation period. These findings indicate durable therapeutic effects post-intervention (Table 1).

**Table 1:** Headache symptom development during the two intervention periods and follow-up.

Period	Headache attack time	Symptoms	Treatments
Before taVNS treatment	Two to three episodes/week; lasting 24 h; mean VAS 5 points; peak at 8–9 points	Phonophobia, photophobia, nausea, vomiting, abdominal distension, and sleep disorders	Motrin 8 mL
Acute intervention period (W1–W4)	One to two episodes/week; mean duration of 225 min; mean VAS 4.2 points	Nausea and vomiting only once, without photophobia	Motrin 8 mL (average 1 dose/week)
Intermission period	Two episodes/week; duration of 300–360 min	No nausea, vomiting, phonophobia, or photophobia	Motrin 8 mL (average 1 dose/week)
Preventive intervention period (W1–W12)	<1 episode/week; mean duration of 234 min; the duration of the last headache was 30 min	No nausea, vomiting, phonophobia, photophobia, or abdominal distension, and sleep disorders were improved	Motrin 8 mL 8 doses in total (<1 dose/week); reduced to 5 mL in the 8th week
Follow-up (W1–W2)	One to two episodes/week; duration of 120–180 min; VAS 4–5 points	No accompanying symptoms	Motrin 5 mL; 2 doses in total
Follow-up (W3–W4)	One episode/week; duration of 30–60 min	No accompanying symptoms	Motrin 5 mL; 1 dose only
Follow-up (W5–W8)	Three episodes in total; duration less than 30 min	No accompanying symptoms	No drugs

## 4 Discussion

This case report describes an 8-year-old boy with refractory chronic migraine with aura who experienced clinically meaningful improvement in attack duration, frequency, aura burden, and rescue medication use following a phased taVNS protocol. Notably, the patient transitioned from frequent, long-lasting, moderate–severe attacks to infrequent, brief, mild attacks at follow-up, with sustained absence of adverse events and elimination of preventive pharmacotherapy.

Non-invasive neuromodulation modalities, including transcranial magnetic stimulation, transcranial electrical stimulation, remote electrical neuromodulation, and nVNS, are attracting increasing attention in migraine management. A growing body of clinical evidence now supports the safety and efficacy of taVNS for adult migraine treatment (15, 16, 21–25). Given its non-pharmacological, peripherally targeted mechanism, taVNS exhibits a favorable safety profile with minimal side effects and a good cognitive tolerability profile (26–28). This may minimize concerns regarding cognitive impairment, which has been observed with some preventive medications (e.g., topiramate) (27).

This case provides early evidence that a phased acute and preventive taVNS strategy may benefit pediatric patients with refractory chronic migraine. TaVNS may represent a viable non-pharmacological alternative for adolescents who experience insufficient relief from standard treatments. The observed reduction in headache frequency and intensity aligns with emerging adult studies (29–31). Aura resolution and reduced medication reliance enhance the clinical relevance of this treatment. Notably, the patient reported better sleep, consistent with taVNS's effects on insomnia (32, 33), suggesting taVNS may simultaneously target migraine comorbidities.

However, interpreting improvements in a single pediatric chronic migraine case requires caution, given the high placebo effects documented in pediatric pain and migraine prevention trials. Contextual factors (expectancy, increased monitoring, and natural fluctuation) could have influenced the observed trajectory. The absence of a sham control, mechanistic biomarkers, and longer follow-up further constrains causal inference and generalizability.

In conclusion, this case supports taVNS as a feasible, safe, and potentially effective modality for both acute and preventive regulation in pediatric chronic migraine with aura. Future work should implement sham-controlled randomized trials, with adequate baseline run-in and standardized outcomes to validate these findings.

## Data Availability

The original contributions presented in the study are included in the article/Supplementary Material, further inquiries can be directed to the corresponding author.
